# Correlations of altered functional connectivity in resting-state fMRI and symptom severity in tic disorders

**DOI:** 10.1192/j.eurpsy.2024.1284

**Published:** 2024-08-27

**Authors:** J. B. Meeh, L. Orth, D. Leiding, U. Habel, I. Neuner, P. Sarkheil

**Affiliations:** ^1^University of Muenster, Muenster; ^2^RWTH Aachen University, Aachen; ^3^Research Center Jülich, Jülich, Germany

## Abstract

**Introduction:**

Vocal and motor tics are characteristic for Tic disorders (TD) and Tourette’s syndrome (APA 2022) . Because of the pathophysiology of the disorders not being fully understood and the presence of the externally measurable symptoms; great attention has been paid to the cortico-striatal regions of patients with TD . In addition to the alterations in motor symptoms patients can experience a premonitory urge (PU) which can be felt before a tic (Reese *et al.* Behav. Ther. 2014; 45 177–186) . Previous studies found an impact of these urges on sensory perception, attention and social cognition as well as an involvement of the brain regions insula, anterior cingulate cortex (ACC) and the temporoparietal junction (TPJ) (Seeley J. Neurosci. 2019; 39 9878–9882, Kucyi *et al.* J. Neurophysiol. 2012; 108 3382–3392, Uddin *et al.* Brain Topogr. 2019; 32 926–942) . These findings lead to the idea of altered functional connectivity of the salience network (SN) in patients with TD.

**Objectives:**

This study aims to investigate the connectivity changes of the SN in patients with TD. We examined functional resting-state scans of patients with TD and searched for possible correlations between the tic and PU severity and the connectivity of the SN.

**Methods:**

21 Patients (mean age: 30.9 years ± 10.0 [range = 19–57], 6 females) diagnosed with TD, and 20 healthy controls (mean age: 29.7 years ± 8.9 [range = 18–50], 5 females) underwent a resting-state fMRI scan. Functional and anatomical images were conducted on a 3T Siemens Prisma fit MRI scanner. PU and tic severity were measured by the Premonitory Urges for Tics Scale (PUTS) and the Yale Global Tic Severity Scale (YGTSS). The connectivity analysis of the resting-state scans was done using the CONN toolbox v21.a. After pre-processing and de-noising steps, a whole-brain seed-based connectivity analysis was carried out with the seeds being the major cortical nods of the SN. For the correlation analysis a linear regression of the YGTSS score/PUTS score and the brain connectivity of the seed regions was conducted.

**Results:**

The PUTS score was 25.3±5.4 (range 10-33) and the YGTSS total tic score was 23.1±7.9 (range 10-38) for the patients. The connectivity analysis revealed a significant difference in connectivity between the groups for the ACC, the right insula and the TPJ. A negative correlation between the YGTSS scores and the connectivity of the left insula and the right superior frontal gyrus (SFG) was shown in the correlation analysis. No significant correlation was found for the PUTS scores in the investigated seed regions.

**Conclusions:**

The right SFG mediates motor urgency and inhibitory control. Since we found a negative correlation between the insula and the right SFG regarding to higher YGTSS scores of the patients, our results might shed some light on the pathophysiology regarding lower inhibitory control in patients which experience higher tic severity.

**Disclosure of Interest:**

None Declared

